# Evaluation of MIC Strip Isavuconazole Test for Susceptibility Testing of Wild-Type and Non-Wild-Type Aspergillus fumigatus Isolates

**DOI:** 10.1128/AAC.01659-16

**Published:** 2016-12-27

**Authors:** Maiken Cavling Arendrup, Paul Verweij, Henrik Vedel Nielsen

**Affiliations:** a Unit of Mycology, Statens Serum Institut, Copenhagen, Denmark; b Department of Clinical Microbiology, Rigshospitalet, Copenhagen, Denmark; c Department of Clinical Medicine, University of Copenhagen, Copenhagen, Denmark; d Department of Medical Microbiology, Radboud University Medical Center, Nijmegen, The Netherlands

**Keywords:** antifungal susceptibility testing, MIC, isavuconazole, gradient strip, EUCAST, Aspergillus fumigatus, Cyp51A mutants, wild type

## Abstract

We evaluated the MIC Strip Isavuconazole test against EUCAST E.Def 9.3 by using 40 wild-type and 39 *CYP51A* mutant Aspergillus fumigatus strains. The strip full inhibition endpoint (FIE) and 80% growth inhibition endpoint were determined by two independent readers, reader 1 (R1) and R2. The essential (within ±0, ±1, and ±2 twofold dilutions) and categorical agreements were best with the FIE (for R1/R2, 42%/41%, 75%/73%, and 90%/89% for essential agreement, and 91.1%/92.4% categorical agreement, with 6.3/8.9% very major errors and 0/1.3% major errors, respectively). The MIC Strip Isavuconazole test with the FIE appears to be useful.

## TEXT

Antifungal susceptibility testing of Aspergillus fumigatus has become increasingly important with the emergence of azole resistance (1–6). EUCAST has set clinical breakpoints for isavuconazole and Aspergillus (7). For A. fumigatus, the clinical breakpoint is 1 mg/liter, one step lower than the epidemiological cutoff value (ECOFF) (2 mg/liter) because the pharmacokinetic/pharmacodynamic breakpoint is 1 mg/liter and the MIC ranges for wild-type and resistant mutants overlap. Hence an MIC of 2 mg/liter may represent wild-type isolates as well as isolates with clinically relevant resistance mechanisms (1–3, 5, 8–15). In clinical practice, the adoption of a restrictive clinical breakpoint for interpretation of MICs generated by commercial tests may create a higher risk of misclassification unless the susceptibility test is very well standardized against the reference method and associated with low reader-to-reader and interlaboratory variations. An isavuconazole gradient strip (Etest; AB Biodisk, Solna, Sweden) was previously evaluated but is no longer available (16, 17). Thus, we evaluated the only commercially available isavuconazole susceptibility test, the MIC Strip Isavuconazole test (Liofilchem, Roseto degli Abruzzi, TE, Italy).

Forty wild-type and 39 *CYP51A* mutant A. fumigatus isolates with hot-spot alterations involving G54 (*n* = 10), M220 (*n* = 10), TR_34_/L98H (*n* = 9), and TR_46_/Y121F T289A (*n* = 10) were included. For the strip test (Liofilchem, Roseto degli Abruzzi, TE, Italy) a McFarland 0.5 conidial suspension and RPMI 1640 2% glucose agar (SSI Diagnostica, Hillerød, Denmark) were used. Strip MICs were read by two independent technicians (reader 1 [R1] and R2) blind to the *CYP51A* genotype at 24 and 48 h of incubation, with an 80% inhibition endpoint (80% IE) and a full inhibition endpoint (FIE). EUCAST testing was performed as previously recommended (7, 18). Four control strains were included (see Table S1 in the supplemental material) (7). The percent essential agreement between the tests was calculated. Isolates for which the MICs were above scale by both methods (EUCAST, >16 mg/liter; strip test, >32 mg/liter) were considered in agreement within ±0 twofold dilution. The categorical agreement between the methods was calculated as the percentage of isolates classified equally by both methods. Very major errors (VMEs) were defined as isolate categorization as resistant (R) by EUCAST but susceptible (S) by the strip test, and major errors (MEs) were defined as isolate categorization as S by the EUCAST method but R by the strip test.

Most isavuconazole strip MICs were above the recommended ranges for the two control Candida strains (Table S1). In contrast, the strip MICs for A. fumigatus ATCC 204305 and A. flavus CM1813 were within ±1 twofold dilution of the EUCAST MICs, suggesting better agreement for the Aspergillus strains and best when using the FIE for Aspergillus.

**FIG 1:**
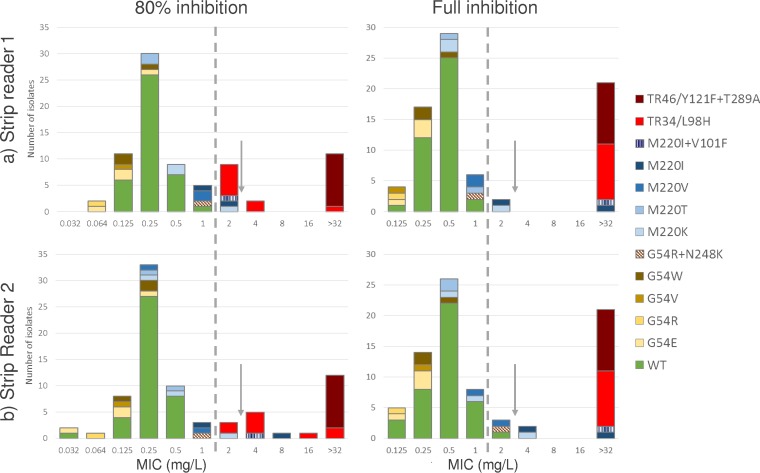
Isavuconazole strip MICs for wild-type and *CYP51A* mutant A. fumigatus isolates determined at 80% inhibition (left side) and full inhibition endpoints (right side) and by two independent readers, R1 (top) and R2 (bottom).

Nine isolates (11.4%) failed to grow sufficiently well to allow strip MIC reading on day 1, when, in general, zones were fuzzy and difficult to read. Day 2 MICs were lower with the 80% IE than with the FIE (Fig. 1). This was particularly evident for isolates harboring TR_34_/L98H alterations, for which the modal 80% IE MICs were 2 and 4 mg/liter, respectively but >32 mg/liter for both readers with the FIE. The essential agreement between the strip MICs from the two readers was highest, 97% at ±1 twofold dilution and 100% at ±2 twofold dilutions, when using the FIE (Table 1).

**TABLE 1:** Essential agreement between R1 and R2 of MIC Strip Isavuconazole test and between strip and EUCAST MICs

CYP51A profile	*n*	Strip MIC agreement (%)^*a*^ between R1 and R2						Agreement (%)^*a*^ between Strip MIC and EUCAST MIC											
		R1 vs R2, FIE			R1 vs R2, 80% IE			R1 FIE vs EUCAST			R2 FIE vs EUCAST			R1 80% IE vs EUCAST			R2 80% IE vs EUCAST		
		±0^*b*^	±1	±2	±0	±1	±2	±0	±1	±2	±0	±1	±2	±0	±1	±2	±0	±1	±2
Wild type	40	55	95	100	83	98	98	38	80	98	28	78	95	18	63	88	15	63	88
G54 alterations																			
G54E	4	100	100	100	75	100	100	75	100	100	75	100	100	0	100	100	0	75	100
G54R	1	100	100	100	100	100	100	0	0	100	0	0	100	0	0	0	0	0	0
G54V	1	0	100	100	100	100	100	0	100	100	100	100	100	0	100	100	0	100	100
G54W	3	100	100	100	67	100	100	33	100	100	33	100	100	67	100	100	100	100	100
G54R N248K	1	0	100	100	100	100	100	0	100	100	100	100	100	0	100	100	0	100	100
M220 alterations																			
M220K	3	33	100	100	67	100	100	0	100	100	67	100	100	0	100	100	0	67	100
M220T	2	50	100	100	50	100	100	100	100	100	50	100	100	0	50	100	50	50	100
M220V	2	50	100	100	50	50	100	0	50	100	50	50	100	0	50	100	0	50	50
M220I	2	50	100	100	50	50	100	50	50	50	0	50	50	0	50	100	50	100	100
M220I V101F	1	100	100	100	0	100	100	0	0	100	0	0	100	0	0	0	0	0	100
Mutants with TR^*c*^																			
TR_34_/L98H	9	100	100	100	33	78	89	11	11	33	11	11	33	11	22	100	33	67	100
TR_46_/Y121F T289A	10	100	100	100	100	100	100	100	100	100	100	100	100	100	100	100	100	100	100
All isolates	79	68	97	100	75	94	97	42	75	90	41	73	89	25	66	91	30	70	91

a Percent essential agreement within ±0, ±1, and ±2 twofold dilutions, respectively. MICs were read after 2 days of incubation. Strip MICs were read by using the FIE or 80% IE endpoint.

b Number of twofold dilutions.

c TR, tandem repeat in the *CYP51A* promoter region.

Isavuconazole MICs for isolates with wild-type *CYP51A* or single alterations at the G54 codon were all below the EUCAST ECOFF for the strip test with the FIE, as well as for EUCAST (Table 2). Likewise, the MICs for isolates harboring M220I alterations or TR_34_/L98H or TR_46_/Y121F T289A were all above the clinical breakpoint for both methods when the FIE was used for the strip test. However, the MICs for TR_34_/L98H isolates were higher when determined by the strip test (MIC range, >32 mg/liter) than when determined by EUCAST (MIC_50_ of 8 mg/liter; range, 4 to >16 mg/liter) (Table 2). The overall essential agreement between strip MICs and EUCAST MICs within ±0, ±1, and ±2 twofold dilutions was best when using the FIE (R1/R2: 42/41, 75/73, and 90/89%) than when using the 80% IE (R1/R2: 25/30, 66/70, and 91/91%). At least 95% essential agreement between the strip test and EUCAST within ±2 twofold dilutions was seen for all *CYP51A* genotypes except those harboring the TR_34_/L98H mechanism or the M220I alteration. Similarly, the categorical agreement was better for the FIE reading of the strip test (91.1 to 92.4% with 6.3 to 8.9% VMEs and 0 to 1.3% MEs) than for the 80% IE (89.9% with 10.1% VMEs and 0% MEs for both readers). VMEs included four isolates with the wild-type *CYP51A* genotype and one to four isolates harboring M220V, M220I, or G54R N248K alterations, respectively.

**TABLE 2:** Isavuconazole susceptibility of wild-type and *CYP51A* mutant A. fumigatus isolates determined by strip test^*a*^ and EUCAST E.Def 9.3

CYP51A profile^*a*^	No. of isolates	EUCAST E.Def 9.3				Gradient strip R1				Gradient strip R2			
		MIC_50_^*b*^	MIC range	% >ECOFF^*c*^	% R	MIC_50_^*b*^	MIC range	% >ECOFF	% R	MIC_50_^*b*^	MIC range	% >ECOFF	% R
Wild type	40	0.5	0.25–2	0	10	0.5	0.125–1	0	0	0.5	0.125–2	0	3
G54 alterations													
G54E	4		0.125–0.25	0	0		0.125–0.25	0	0		0.125–0.25	0	0
G54R	1		0.5	0	0		0.125	0	0		0.125	0	0
G54V	1		0.25	0	0		0.125	0	0		0.25	0	0
G54W	3		0.125–0.25	0	0		0.25–0.5	0	0		0.25–0.5	0	0
G54R N248K	1		2	0	100		1	0	0		2	0	100
M220alterations													
M220K	3		1–4	33	33		0.5–2	0	33		0.5–1	33	33
M220T	2		0.5–1	0	0		0.5–1	0	0		0.5	0	0
M220V	2		2–4	50	100		1	0	0		1–2	0	50
M220I	2		2–8	50	100		2 to >32	50	100		4 to >32	100	100
M220I V101F	1		16	100	100		>32	100	100		>32	100	100
Mutants with TR^*d*^													
TR_34_/L98H	9		4 to >16	100	100		>32	100	100		>32	100	100
TR_46_/Y121F T289A	10	>16	>16	100	100	>32	>32	100	100	>32	>32	100	100
All isolates	79	1	0.125 to >16	29	38	0.5	0.125 to >32	27	29	0.5	0.125 to >32	29	33

a Plates were read by two independent readers using the FIE endpoint after 2 days of incubation.

b MIC_50_s (mg/liter) are presented only for genotypes represented by ≥10 isolates.

c Percentage of isolates with MICs above the EUCAST isavuconazole ECOFF (2 mg/liter) and clinical (1 mg/liter) breakpoints.

d TR, tandem repeat in the *CYP51A* promoter region.

The MIC Strip Isavuconazole test manufacturer recommends an 80% IE reading, but in this study, higher interreader essential agreement, better separation between wild-type and resistant strains, and greater essential and categorical agreement compared to EUCAST results were achieved with the FIE. Thus, the FIE criterion was found to be superior although the MICs for the recommended Candida control strains were out of range (7). When using the FIE, the essential agreements with EUCAST within ±1 and ±2 twofold dilutions were 73 to 75% and 89 to 90% and thus better than previously found for the isavuconazole Etest versus the CLSI method, even though challenged here with a strain collection including a significant number of non-wild-type isolates (16). The categorical agreement was >91% when interpreting the MICs according to EUCAST breakpoints, and notably, among the 6 to 9% VMEs, half were isolates with a wild-type *CYP51A* target gene that either may be harboring other resistance mechanisms or may be isolates that are truly susceptible but misclassified as R by the EUCAST reference method because of the conservative EUCAST susceptibility breakpoint (7). Finally, the separation between wild-type and TR_34_/L98H and TR_46_/Y121F T289A mutant isolates was greater for the MIC strip test, rendering it a potentially promising routine lab tool for detecting R environmental mutants, provided the FIE is used (2, 5, 19–21).

The CYP51A amino acid alterations have been associated with a codon-specific susceptibility pattern (4, 13). Here, both the strip and EUCAST isavuconazole MICs indeed straddled the clinical breakpoint for isolates harboring M220 alterations and for the G54R N24K double mutant, which will inevitably lead to the random classification of such isolates as S or R in routine testing. Hence, as long as clinical outcome data are unavailable for such mutants, other measures such itraconazole MIC testing or *CYP51A* sequencing should be undertaken to detect these genotypes.

This study has limitations. We investigated strip test reader-to-reader agreement but no other factors associated with variation, such as variation across different lots and brands of RPMI agar plates, inoculum preparation, etc. Therefore, the promising performance reported here needs confirmation in a multicenter study.

## Supplementary Material

Supplemental material
