# Supplementary material for: Evaluation of MIC Strip Isavuconazole Test for Susceptibility Testing of Wild-Type and Non-Wild-Type Aspergillus fumigatus Isolates
Source: Antimicrob Agents Chemother. 2016 Dec 27;61(1):e01659-16. doi: 10.1128/AAC.01659-16 (PMC5192160; doi:10.1128/AAC.01659-16)
Supplement: Supplemental material [file supp_61_1_e01659-16__index.html]

Evaluation of MIC Strip Isavuconazole Test for Susceptibility Testing of Wild-Type and Non-Wild-Type Aspergillus fumigatus Isolates — Supplemental material 

# Evaluation of MIC Strip Isavuconazole Test for Susceptibility Testing of Wild-Type and Non-Wild-Type Aspergillus fumigatus Isolates

## Supplemental material

- Supplemental file 1 -

  Table S1

  PDF, 317K
