# Supplementary material for: Evaluation of MIC Strip Isavuconazole Test for Susceptibility Testing of Wild-Type and Non-Wild-Type Aspergillus fumigatus Isolates
Source: Antimicrob Agents Chemother. 2016 Dec 27;61(1):e01659-16. doi: 10.1128/AAC.01659-16 (PMC5192160; doi:10.1128/AAC.01659-16)
Supplement: Supplemental material [file AAC.01659-16_zac001175829s1.pdf]

Supplementary Table 1. Isavuconazole strip and EUCAST MICs obtained in this study for quality control strains. Recommended EUCAST MIC ranges are included for the *Candida* control strains but have not yet been established for the *Aspergillus* control strains.

	Incubation (days)	Strip MIC Range (mg/L)		EUCAST MIC (mg/L)	EUCAST QC Reference range (mg/L)
		Full inhibition[a]	80% inhibition[a]		
C. parapsilosis ATCC22019	1	0.03-**0.06**	0.03	≤ 0.015	0.008-0.03
C. krusei ATCC6258	1	**0.25-0.5**	**0.125-0.25**	0.015	0.015-0.06
A. fumigatus ATCC204305	1	0.25-0.5	0.125-0.25	ND[b]	NA[c]
A. flavus CM1813	1	0.5-1	0.125-0.5	ND[b]	NA[c]
A. fumigatus ATCC204305	2	0.25-0.5	0.25	0.5-1	NA[c]
A. flavus CM1813	2	0.5-1	0.25-0.5	0.5-2	NA[c]

[a] The gradient strip MICs were read using a full inhibition endpoint (clear zone) and a less stringent endpoint of 80% inhibition. Bold underlined ranges indicate values outside the recommended EUCAST QC range.

[b] ND: not done

[c] NA: not available (EUCAST has not yet established quality control MIC ranges for the given species). However, isavuconazole EUCAST day 2 MICs for *A. fumigatus* ATCC204305 and *A. flavus* CM1813 were in agreement with the wild type susceptibility pattern for these species (*A. fumigatus*: modal MIC 0.5 mg/L, ECOFF 2 mgland *A. flavus* Modal MIC 1 mg/L, ECOFF 2 mg/L, respectively), suggesting acceptable performance also for *Aspergillus*.
